# Continuous theta-burst stimulation enhances and sustains neurogenesis following ischemic stroke

**DOI:** 10.7150/thno.71832

**Published:** 2022-07-18

**Authors:** Xuemei Zong, Jie Gu, Siqi Zhou, Ding Ding, Yuting Hu, Lorelei Tucker, Zhihai Huang, Deqin Geng, Dianshuai Gao

**Affiliations:** 1 Nanjing Medical University, Nanjing, 211166, China; 2 Emergency Center, the Affiliated Hospital of Xuzhou Medical University, Xuzhou, 221002, China; 3 Department of Neurology, the Affiliated Hospital of Xuzhou Medical University, Xuzhou, 221002, China; 4 Department of Anatomy and Neurobiology of Xuzhou Medical University, Xuzhou, 221004, China; 5 Augusta University, Augusta, Georgia, 30912, USA

**Keywords:** Ischemic stroke, Continuous theta-burst stimulation (cTBS), Neurogenesis, Functional recovery, Neurotrophic factors

## Abstract

**Rationale:** Previous work has indicated that continuous theta-burst stimulation (cTBS), a modality of transcranial magnetic stimulation (TMS), may provide neuroprotection and improve neurological function after stroke by preserving the blood-brain barrier, altering glial polarization phenotypes, and supporting peri-infarct angiogenesis. The present study was performed to examine whether cTBS, a noninvasive neurostimulation technique, promotes neurogenesis in a photothrombotic (PT) stroke rat model and contributes to functional recovery.

**Methods:** Beginning 3 h or 1 week after the induction of PT stroke, once-daily 5-min cTBS treatments were applied to the infarcted hemisphere for 6 days. Samples were collected 6 days, 22 days, and 35 days after PT stroke. Fluorescent labeling, Western blotting, and behavioral tests were performed accordingly.

**Results:** We found that cTBS therapy significantly expanded the pool of neural progenitor cells (NPCs) and newly generated immature neurons in the cortical peri-infarct region after PT stroke. Likewise, the amount of DCX-positive immature neurons in the peri-infarct area was markedly elevated by cTBS. Application of cTBS strikingly diminished the PT-induced loss of NPCs and newly-formed neurons. In addition, the amount of newly generated mature neurons in the peri-infarct zone was significantly promoted by cTBS. Intriguingly, cTBS reduced reactive gliogenesis significantly while promoting oligodendrogenesis and preserving myelination. Mechanistic studies uncovered that cTBS upregulated brain-derived neurotrophic factor (BDNF) and fibroblast growth factor 2 (FGF2). Finally, cTBS-treated animals displayed improved motor functions. To be noted, temozolomide (TMZ), a drug that has been previously used to suppress neurogenesis, could reverse the beneficial effects of cTBS.

**Conclusions:** Our findings provide new insight into the mechanism by which cTBS promotes functional recovery from stroke. We demonstrated that cTBS effectively enhances and sustains neurogenesis after PT stroke. Both early and delayed cTBS treatment could improve the survival of newly generated neurons and functional recovery, and inhibition of neurogenesis could reverse these therapeutic benefits. Mechanistically, cTBS was effective in upregulating the release of neurotrophic factors, protecting NPC and immature neurons, as well as suppressing excessive gliogenesis.

## Introduction

Stroke, the cessation or reduction of blood flow to a part of the brain, is a pressing public health issue in the United States, affecting nearly 800,000 people every year 1. This disease takes a drastic toll on patients; ~150,000 people died from a stroke in 2018 alone, and 50% of survivors developed some form of chronic disability 1, 2. Ischemic stroke is the most common form of stroke, accounting for 87% of cases, and occurs when a cerebral blood vessel is occluded, usually from a clot. Unfortunately, effective treatment options for stroke are currently limited to mechanical thrombectomy and tissue plasminogen activator (tPA) 3. However, both of these treatment strategies must be applied within a short time window to save brain tissue within the core infarct, 4.5 hours for tPA and 6 hours for mechanical thrombectomy 4. As a result, only a few patients will benefit from these treatment interventions, and the functional recovery of survivors is usually limited. Indeed, even if reperfusion means such as intravenous thrombolysis and endovascular therapy are applied, the 90-day good prognosis rate (mRS score 0-2) of patients is only about 50%, with a high recurrence rate of related symptoms. Hence, there is an urgent need to develop new treatment strategies that can effectively reduce disability rates and facilitate functional recovery.

The process of post-stroke neuronal cell death is not immediate, however. While the tissue at the infarct site is lost due to necrotic cell death within four hours of stroke, neurons in the surrounding tissue, called the penumbra or peri-infarct region, slowly die over the next few days through the process of programmed neuronal cell death 5. Thus, if the peri-infarct tissue could be preserved or restored, neurological outcomes for patients could be improved. Therefore, therapies promoting endogenous repair mechanisms are highly sought after and are the goal of extensive biomedical research.

Once thought to be a phenomenon limited to early development, adult neurogenesis has since been well-established as occurring in response to ischemic brain injury 6-8. Shortly after cerebral ischemia, neural progenitor cells (NPCs) from the subventricular zone (SVZ) and the subgranular zone (SGZ) begin proliferating and migrating to the peri-infarct area 9. Once there, they begin to differentiate and attempt to incorporate themselves into the neuronal architecture. Unfortunately, the harsh post-stroke neuronal microenvironment takes a toll on NPCs and newly formed neurons, killing most of them long-term 10, 11. The hostile milieu present after stroke is mediated, in part, by activated astroglial cells 12.

Activated astrocytes release inflammatory factors that can, in the early phase, stimulate a neurogenic response. However, a chronic neuroinflammatory response creates toxic microenvironmental conditions that are harmful to new neurons 13-15. However, astroglia can release anti-inflammatory factors and trophic factors such as brain-derived neurotrophic factor (BDNF) and fibroblast growth factor 2 (FGF2) that are critical for supporting new neurons and oligodendrocyte progenitors as they attempt to repair the peri-infarct site 13, 14, 16, 17. Thus, one key to neural repair may be noninvasive treatments like transcranial magnetic stimulation (TMS) that target different components that contribute to the neuronal microenvironment 18.

Continuous theta-burst stimulation (cTBS) is a modality of TMS that has shown many beneficial actions in the context of stroke and other brain injury models 19. In TMS, a strong magnetic field is applied to a specific brain region to induce a current that can either stimulate or suppress local activity 19. This noninvasive therapy is currently used in the clinic for treatment-resistant depression and is praised for its efficacy and minimal adverse effects 20. In our previous work, we applied cTBS to the photothrombotic stroke model in rats, which uses a photochemical reaction to generate a core infarct surrounded by a rim of salvageable peri-infarct tissue 18, 21. We found that cTBS preserved neuronal survival and functional outcomes by ameliorating the hostile post-stroke microenvironment. Furthermore, astroglial and microglial phenotype polarization was shifted from the damaging proinflammatory states to a beneficial anti-inflammatory phenotype in cTBS-treated rats. In addition, cTBS stimulated angiogenesis in the peri-infarct region, which is spatiotemporally coupled with neurogenesis 21, 22. Therefore, we conducted the current study to investigate if cTBS can stimulate and support neurogenesis after experimental stroke and determine whether this is supported by trophic factor release.

## Materials and Methods

### Photothrombotic (PT) Stroke Model

Adult 3-month-old male Sprague-Dawley rats were randomly assigned into the following 3 groups: (1) sham control group without PT stroke and with sham cTBS treatment (Ctl group), (2) PT stroke group with sham cTBS treatment (PT group), and (3) experimental group with PT stroke and cTBS treatment (cTBS group). The photo-irritated PT stroke model was carried out according to the protocol as we described recently 18, 21. Briefly, sodium pentobarbital (50 mg/kg body weight) was intraperitoneally injected to anesthetize the rats, and the animals were placed in a stereotactic frame. The scalp was incised along the midline and hydrogen peroxide was used for cleaning the skull surface. Cold-light beam of 6 mm diameter (Zeiss Schott KL 1500 Electronic Microscope Light Source, 150W, Carl Zeiss, Germany) was guided through a fiber optic cable and located closely in touch to the skull surface. The light spot center was aimed at 1.8 mm anterior to the bregma and 2.5 mm lateral from the midline. Rose Bengal dye (0.1 mg/g body weight, i.p.) was administrated by intraperitoneal injection and allowed to circulate in the rat for 5 min, and then the skull was irradiated with cold light for 15 min. Afterwards, the site of incision was cleaned with Betadine and the skin was sutured and the animals were returned to their cages. Rectal temperature was monitored and maintained at 36.5-37.5 °C during and after surgical procedures via a heating pad and an overhead infrared lamp. All animal procedures were approved by the local Institutional Animal Care and Use Committee and complied with the guidelines of the National Institutes of Health. Every effort was made to minimize pain or distress for the animals involved.

### Continuous theta-burst stimulation (cTBS) treatment and administration of BrdU and temozolomide

As described previously 18, theta-burst rTMS treatment (3 pulses of 50 Hz, repeated every 200 ms) was administrated for 5 min targeting at the infarcted hemisphere via a pair of Helmholtz coils. This paradigm is typically observed to induce LTD-like plasticity alterations in the motor cortex of human subjects and animals 19, 23. Treatment was initiated 3 h after PT stroke on the surgery day (referred to day 1), and administered daily for 6 consecutive treatments. For cTBS treatment, each single animal was restrained in a transparent DecapiCone, and the infarct brain was exposed to the magnetic field adjusted to 200 Gauss (FW Bell RoHS meter #5180, OECO LLC, OR, USA). In additional experiments, delayed cTBS was applied to the rats one week after PT stroke for 6 days, and the levels of neurogenesis and motor functions were determined 5 weeks after stroke. Sham cTBS-stimulated animals experienced the identical procedure as with cTBS-treated stroke animals, except that the cTBS magnetic field was adjusted to zero. Immediately after cTBS or sham cTBS treatment, BrdU (50 mg/kg body weight) was intraperitoneally administered to each animal over 6 days to evaluate cell proliferation. To suppress adult neurogenesis, temozolomide (TMZ, Sigma) or vehicle (0.9% NaCl) was administrated with 7-day release Alzet osmotic mini-pumps (1007D, DURECT Corporation, Cupertino, CA) at 10 mg/kg/day. The mini-pumps were implanted subcutaneously in the animals on day 8 after PT stroke with delayed cTBS treatment.

### Brain sections and protein preparations

Rats were sacrificed under anesthesia on day 6 (3 h after the last cTBS or sham cTBS treatment), 22 or 35 after PT stroke. After transcardial perfusion with ice-cold saline followed by perfusion of 4% paraformaldehyde (PFA) in 0.1 M phosphate buffer (PB), the rats were decapitated and the whole brains were then quickly removed. Brains were postfixed at 4 °C overnight and then cryoprotected with 30% sucrose. Afterwards, brains were embedded in OCT medium, frozen, and coronal sections were collected on a Cryostat (Leica RM2155, Nussloch, Germany). For protein preparation, brain tissues from the peri-infarct regions and sham controls were dissected from the cortex and immediately frozen in liquid nitrogen. The tissues were homogenized as previously described 18. In brief, the brains were homogenized using a motor-driven Teflon homogenizer in ice-cold homogenization medium (50 mM HEPES, pH 7.4, 150 mM NaCl, 12 mM β-glycerophosphate, 1% Triton X-100) with inhibitors of proteases and enzymes (Thermo Scientific, Rockford, IL). Modified Lowry Protein Assay (Pierce, Rockford, IL) was used for measuring protein concentrations.

### Immunohistochemistry Staining and Confocal Microscopy

The peri-infarct brain area of coronal slices was chosen for imaging and analysis, as we described previously 18. Immunofluorescence staining of free-floating sections was performed following our previous study 18. Briefly, tissue sections were washed with 0.4% Triton X-100 for 3×10 min, followed by blocking with 10% normal donkey serum for 1 h, and incubation overnight with primary antibody at 4 °C. The following primary antibodies were used: anti-DCX, p-TrkB (Tyr816) (Novus Biologicals); anti-Nestin (2Q178), SOX2, Tuj1, NeuN, Olig2, FGF2, BDNF, MBP, BrdU (Thermo Fisher Scientific); anti-Iba1, GFAP (Proteintech). Sections were then washed and incubated with appropriate secondary antibodies conjugated to Alexa Fluor (Thermo Fisher Scientific) for 1 h. After washes, the sections were counterstained and coverslipped in Vectashield mounting medium without or with DAPI (Vector Laboratories). For co-labeling with BrdU, PFA-fixed brain sections were permeabilized sequentially with 1 M HCl for 10 min at 0 °C, 1 M and 2 M HCl for 10 min each at room temperature, then 2 M HCl for 20 min at 37 °C. The sections were neutralized with 0.1 M sodium tetraborate (pH 8.5) for 20 min, followed by washes, blocking, and incubation with anti-BrdU antibody combinations overnight at 4 °C. Tunel staining was performed on free-floating sections using a Click-iT® Plus TUNEL assay kit (Thermo Fisher Scientific), as described in our recent work 18. LSM700 Meta confocal laser scanning microscope (Carl Zeiss, Germany) was used for visualizing and capturing the double and triple-stained sections in XYZ plane using a 40× oil immersion Neofluor objective (512×512 pixels size). The sections were scanned using Z-series scanning (20 optical slices) with 12-bit pixel depth using optimum pinhole diameter. The Z-stacks were then converted into 3D projection images using ZEN imaging software from Carl Zeiss, and representative fluorescent images were presented. For the analyses of double staining labeled cells (Nestin/SOX2, Nestin/Tuj1, DCX/BrdU, DCX/NeuN, SOX2/Tunel, NeuN/BrdU, BrdU/Iba1, BrdU/GFAP, BrdU/Olig2, MBP/DAPI, FGF2/GFAP, p-TrkB/BDNF) and triple labeled endothelial cells (Tunel/BrdU/NeuN), 3-5 representative capture views of the peri-infarct brain areas from each animal were adopted. Data from each animal section were then averaged to provide a single value for the specific group. Nestin area fraction, directionality histograms of Tuj1, and the fluorescence intensity profiles BDNF/p-TrkB were analyzed using Fiji software (ImageJ, NIH, MD, version 1.52q). Volume of DCX clusters was analyzed using the Imaris measurement module (Imaris 9.5.0, Bitplane, Switzerland) following surface rendering of the Z-stacked confocal images. A mean ± SE was calculated from the data in each group and used to determine between groups.

### Western Blotting Analysis

Western blotting assays were conducted as we previously described 18. Firstly, 50 μg proteins were subjected to sodium dodecyl sulfate-poly-acrylamide gel electrophoresis (SDS-PAGE) and transferred to polyvinylidene difluoride (PVDF) membrane. The membranes were blocked for 1 h at room temperature using the blocking buffer with gentle shaking and followed by adding the primary antibody and incubating at 4 °C overnight. The following antibodies were used in the immunoblotting experiments: anti-BDNF, FGF2 (Thermo Fisher Scientific), and β-Actin (Proteintech). Dilution and concentration used were optimized according to the antibody protocol instructions. After washes, the membranes were incubated with horseradish peroxidase (HRP)-conjugated secondary antibodies for 1 h at room temperature. Subsequently, chemiluminescent signals were captured and visualized with a CCD-based imaging system (ImageQuant LAS 4000, GE Healthcare). The Fiji/ImageJ software was used for semi-quantitative analysis. The relative level of protein expression was calculated after normalization to the β-Actin protein. Data were present as means ± SE for graphical presentation and statistical analysis.

### Behavioral Assessments

Neurological behavior assessments including the forelimb placing test and the balance beam test were performed 22 days after PT stroke. The motor deficits were examined by the forelimb placement test as described previously 18. In the current experiment, unilateral damage to the right forelimb region of the rat brain sensorimotor cortex caused deficit in the somatosensory function and use of the contralateral (left) forelimb. Forelimb placing of each forelimb was induced by touching the respective vibrissae on the edge of a table top. Each rat was tested 10 times for each forelimb, and the percentage of trials in which the rat placed the left forelimb on the edge of the countertop was determined. Furthermore, the balance beam test was used for assessing motor balance and coordination as previously described 18. The animals were habituated to the balance beam (length: 100 cm; width: 7 cm; height: 100 cm above the ground) 1 day prior to the testing day. During habituation and testing, the animals were placed at one end of the beam and allowed to cross the beam to return to their home cage. The numbers of missteps were recorded by an overhead camera, analyzed, and compared between groups.

The Novel Object Recognition (NOR) test was used to test learning and recognition memory as previously described 24. Briefly, rats were allowed 5 min to explore the arena of the box (40×50×50 cm) containing two identical objects. One day later, the rats were returned to the same box with one familiar object and a novel object to test their object recognition memory. Exploration of the object was defined as the animal's nose within the 2-cm zone. The time spent exploring each object and the discrimination index (% time spent exploring the new object) were recorded and analyzed using ANY-maze video tracking software.

### Statistical Analysis

All the data were expressed as mean ± SE and data analysis was carried out by SigmaStat 3.5 software (SPSS, Inc., IL, USA). The statistical significance was determined using one-way analysis of variance (ANOVA) with Dunnett's multiple-comparisons post hoc test. *P* < 0.05 was considered as statistically significant.

## Results

### cTBS increased the neural progenitor cell pool and the amount of newly generated immature neurons in the peri-infarct cortical area after PT stroke

**Figure 1:**
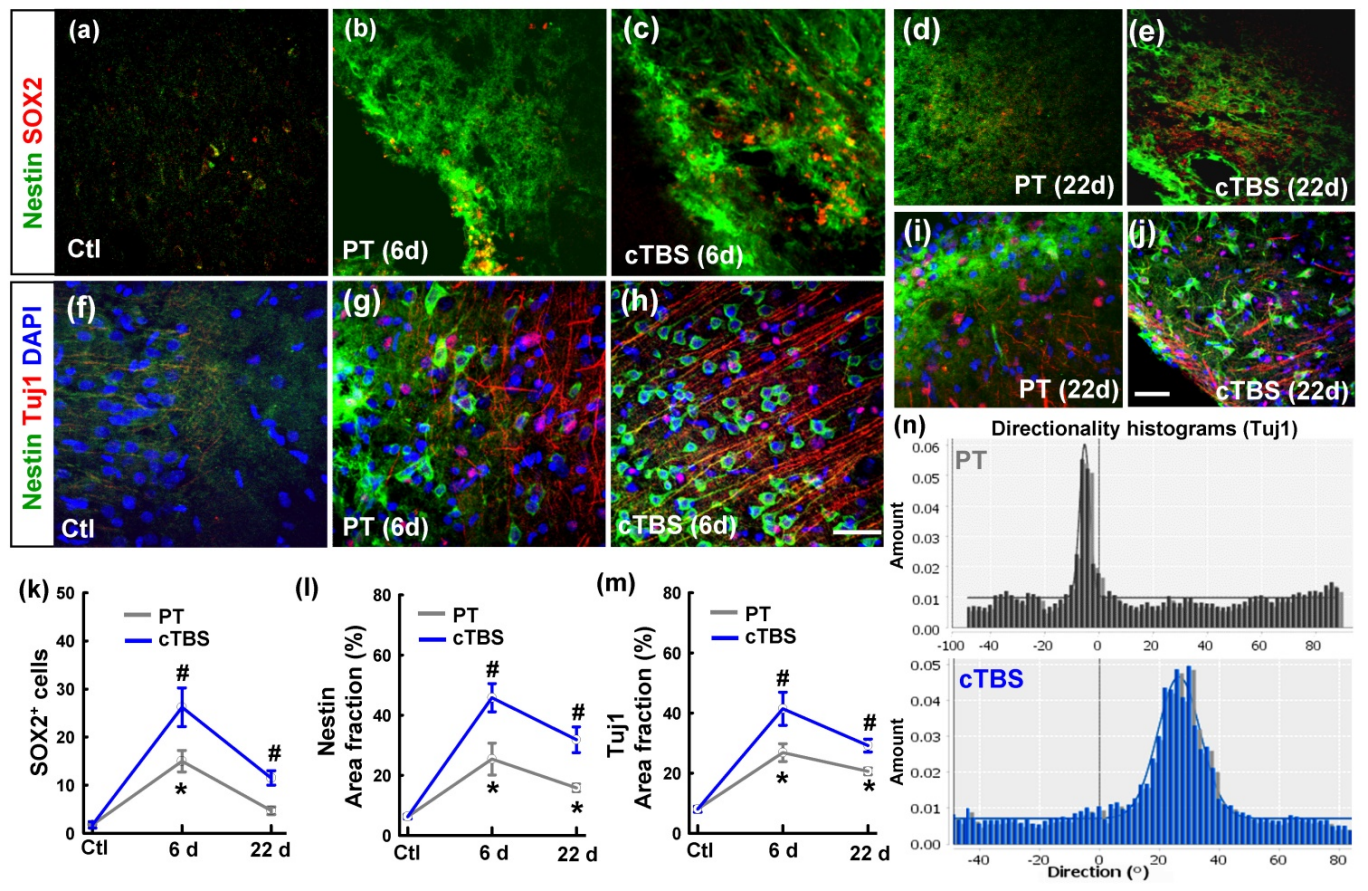
** cTBS increased the neural stem/progenitor cell pool and the level of newly generated immature neurons in the peri-infarct cortical area after PT stroke. (a-j)** Representative micrographs of the two well-described neural stem/progenitor markers (Nestin and SOX2), and a marker of newly generated immature neurons (TuJ1). **(k-m)** Quantitative analyses of SOX2 positive (SOX2^+^) cells, and the area fractions of Nestin and TuJ1 in the peri-infarct region. (n) Representative directionality histograms of Tuj1 in PT group and cTBS group at day 6 after PT stroke. Scale bars = 50 µm, Magnification: ×40. *P < 0.05 versus Ctl group, ^#^*P* < 0.05 versus PT group without cTBS at day 6 and 22 time points, respectively. Data are presented as mean ± SE (N = 5-8).

To identify whether rTMS can boost the neuronal progenitor cell pool and newly formed immature neurons in the peri-infarct region after stroke, we co-stained slices for Nestin/SOX2 **(Figure 1 (a-e))** and Nestin/Tuj1 **(Figure 1 (f-j))** for analysis by confocal microscopy. As shown in the representative images and data analyses** (Figure 1 (k, m))**, PT stroke itself could elevate SOX2 positive cells on day 6 and but not on day 22, compared to sham cTBS treatment (Ctl) group on day 1. PT stroke could also markedly elevate Nestin and Tuj1 positive area in both groups on days 6 and 22, with peak levels on day 6 after stroke. Meanwhile, all the three markers were further enhanced by cTBS compared to the PT group without cTBS at day 6 and 22 time points, respectively. These results indicate that the number of neural progenitors in the peri-infarct area was higher in the cTBS treated animals, and a greater population of immature neurons in the peri-infarct area at these time points. In addition, representative directionality histograms **(Figure 1 (n))** showed increased alignment of Tuj1-positive neuronal processes at day 6 in cTBS animals, suggesting they may be migrating to the peri-infarct area more directly.

### cTBS increased the amount of DCX positive immature neurons in the peri-infarct area 6 days after PT stroke

**Figure 2:**
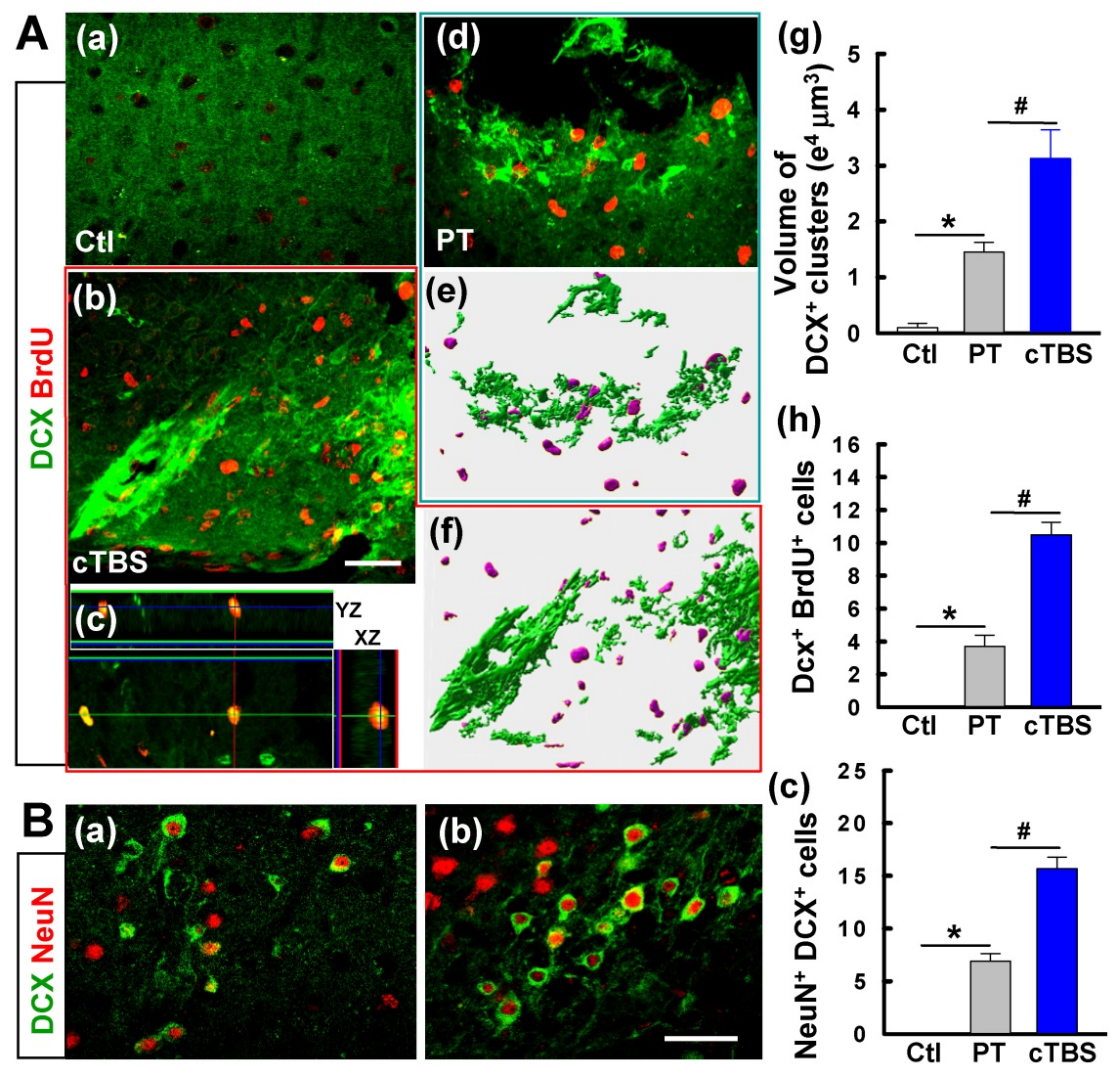
** cTBS increased the level of DCX positive immature neurons in the peri-infarct area 6 days after PT stroke. (A) (a-d)** Demonstrative immunostaining for DCX and BrdU (five daily injections) shows the chains of neuroblasts and individual young neurons in the peri-infarct area. **(e-g)** Representative images from PT and cTBS groups were processed by surface rendering **(e, f)** with Bitplane Imaris and the volume of DCX clusters was analyzed, respectively.** (h)** DCX-positive cells colabeled with BrdU were calculated and compared between groups. **(B) (a-c)** Immunoreactivity of DCX (green), and NeuN (red) in the peri-infarct zone at day 6. Scale bars represent 50 μm. **(c)** Numbers of young neurons (DCX^+^/NeuN^+^) from brains of the PT control and cTBS groups. Data are the mean ± SE (N =5-8). *P < 0.05 versus Ctl group, ^#^*P* < 0.05 versus PT group without cTBS.

Coronal slices from brains 6 days after PT stroke were co-stained for DCX and BrdU to determine if cTBS increased the number of immature neurons in the peri-infarct area. As seen in **Figure 2A**, both PT and cTBS animals had an apparent elevated level of DCX^+^ clusters compared to the control group **(Figure 2A (a-b, d-f))**, as expected after stroke. The cTBS group, however, exhibited a markedly higher amount of DCX^+^ clusters than PT stroke only group. Representative 3D images processed with surface rendering suggest that the volume of DCX^+^ clusters was raised in the cTBS group compared to PT only group **(Figure 2A (e-g))**. Likewise, the numbers of DCX^+^/BrdU^+^ cells in the PT and cTBS groups were significantly higher than control group, but cTBS animals showed more pronounced elevated levels of double-stained immature neurons than the other groups **(Figure 2A (a-d, h))**. In addition, compared to PT group, cTBS-treated stroke animals displayed dramatic elevations in DCX^+^/NeuN^+^ immature neurons **(Figure 2B (a-c))**.

### cTBS treatment attenuated apoptotic cell death of neural progenitor cells and newborn neuronal cells in the peri-infarct region at 6 and 22 days after PT stroke

**Figure 3:**
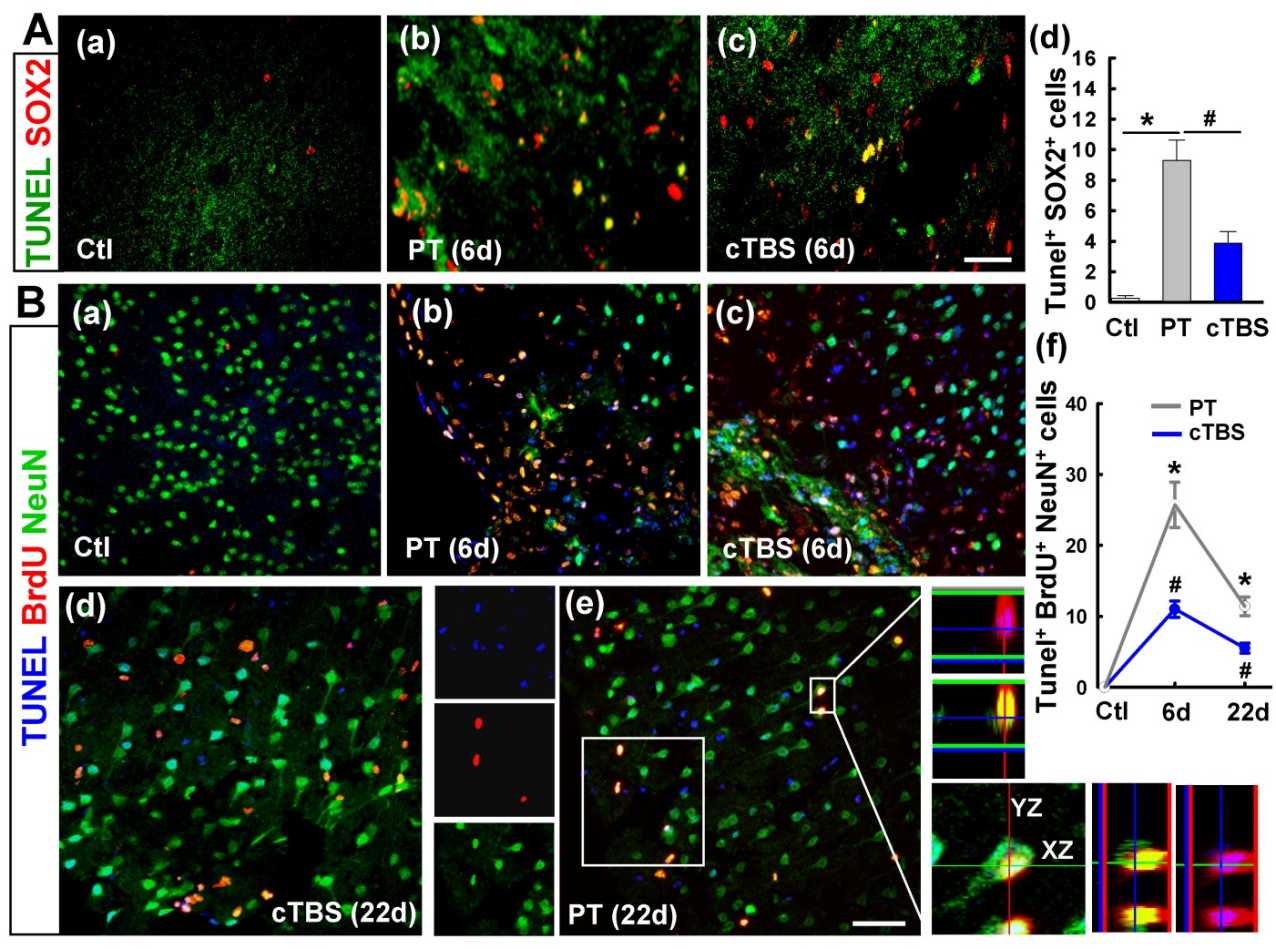
** cTBS treatment attenuated the dead counts of neural progenitor cells and newborn neuronal cells in peri-infarct region examined at 6 and 22 days after PT stroke. (A) (a-d)** Double labeling of brain slices 6 days after PT stroke indicates co-expression between SOX2 and TUNEL (yellow), and the cells were counted. **(B) (a-e)** Representative triple-labeled confocal images of BrdU (red), NeuN (green) and TUNEL (blue) on the sections at 6 and 22 days after PT stroke. Confocal orthogonal view of zoomed image (inset white color) in typical PT group is placed in the right panels, showing the three​-color co-localization **(e)**. The counts of TUNEL positive newborn neurons (BrdU and NeuN double positive cells) were analyzed and shown **(f)**. Scale bars = 50 μm. Data are expressed as mean ± SE (N = 5-8). *P < 0.05 versus Ctl group, ^#^*P* < 0.05 versus PT group without cTBS.

Neuronal progenitor cells are particularly sensitive to the maladaptive microenvironment after stroke and often undergo apoptotic cell death 9. To determine whether cTBS could protect neural progenitor cells, apoptotic cell death of these cells in the peri-infarct region was assessed via TUNEL/SOX2 co-staining and confocal microscopy **(Figure 3A (a-c)).** Analysis of confocal images showed marked cell death in both PT and cTBS groups compared to sham control animals, but it was effectively abated in the cTBS group compared to PT **(Figure 3A (d))**. To further investigate the apoptotic cell death in newborn neuronal cells, the slices were co-stained for TUNEL/BrdU/NeuN. As quantified in **Figure 3B (a-f)**, cell death was higher in both groups on day 6 than on day 22, but cTBS significantly lowered the number of newborn neuronal cell deaths at both time points. Collectively, these data indicate that cTBS treatment effectively protects against apoptotic cell death of neural progenitor cells as well as newborn neuronal cells following stroke.

### cTBS enhanced the counts of newly generated neurons in the peri-infarct zone after PT stroke

**Figure 4:**
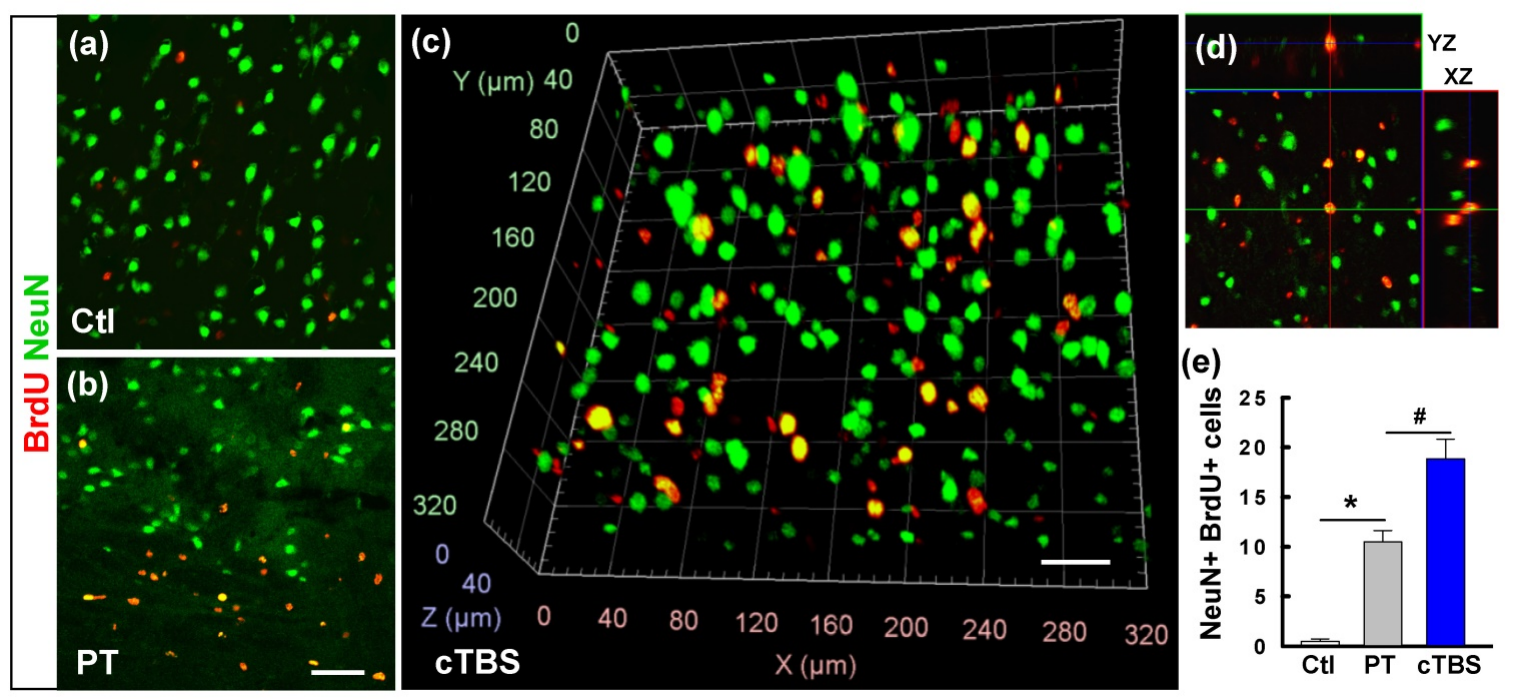
** cTBS enhanced the counts of newly generated neurons in the peri-infarct zone after PT stroke. (a-d)** Representative confocal images in the peri-infarct area of Ctl and PT stroke animals at 22 d from the first BrdU injection. Typical NeuN and BrdU double-labeled cells in the cTBS group were viewed with the aid of arivis Vision4D software **(c)**. Reconstructed orthogonal view of a BrdU (red) and NeuN (green) double-labeled cell, as shown in the y-z and x-z planes (right, **d**). The total numbers of newly generated neurons from the animals in each group were counted and plotted **(e)**. Scale bars = 50 μm. Values indicate mean ± SE (N = 6-8). *P < 0.05 versus Ctl group, ^#^*P* < 0.05 versus PT group.

After ischemic strokes, NPCs existing in the SVZ of the lateral ventricle and SGZ of the dentate gyrus, or in the peri-infarct area, proliferate and migrate toward the lesion, contributing to brain repair. This endogenous neurogenesis, however, is slow and does not lead to fully restored brain function 25. Therefore, it has been proposed that amplifying endogenous neurogenesis may represent a novel therapeutic strategy for stroke 26. To assess the effects of cTBS on the number of newly generated mature neurons in the peri-infarct region after stroke, sections taken on day 22 after PT were co-stained for NeuN/BrdU **(Figure 4 (a-d))**. Analysis revealed that the amount of surviving NeuN^+/^BrdU^+^ neurons was increased following PT stroke **(Figure 4 (e))**. Intriguingly, this effect was enhanced in cTBS animals which had significantly more NeuN^+^/BrdU^+^ cells than the PT group. These findings clearly indicate that cTBS could boost neurogenesis and maintain the survival of newly generated mature neurons in the peri-infarct zone, which may provide the basis for tissue regeneration.

### cTBS treatment attenuated gliogenesis in the peri-infarct zone after PT stroke

**Figure 5:**
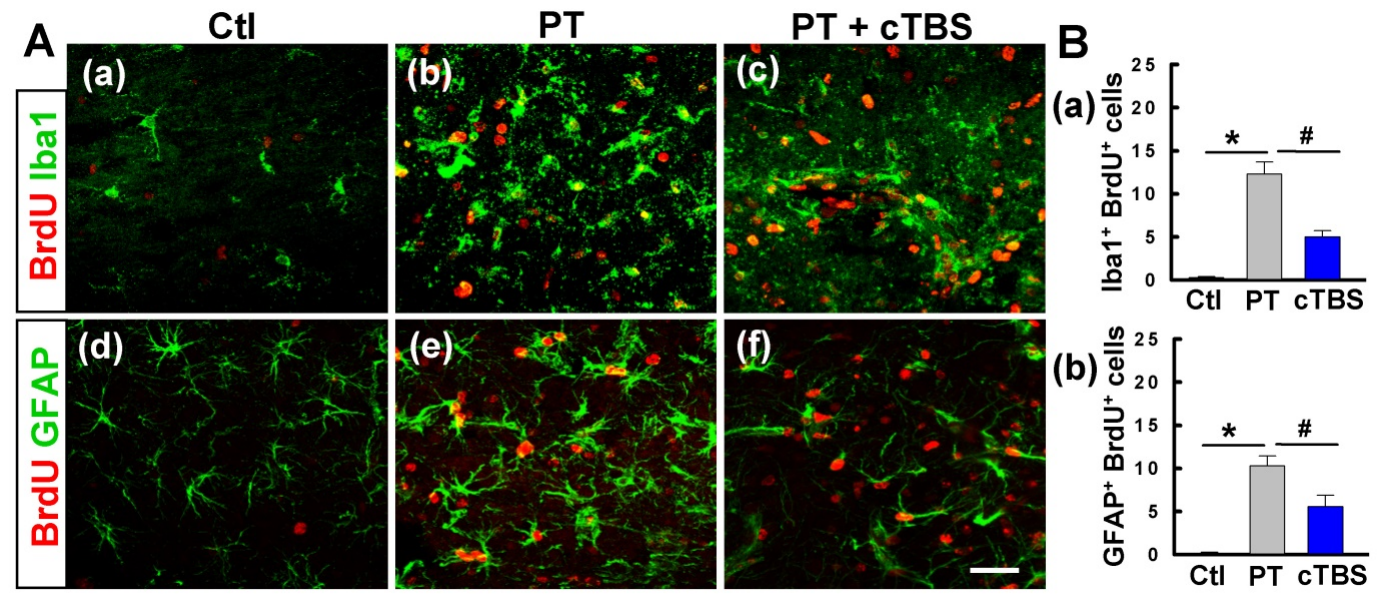
** cTBS treatment attenuated gliogenesis in the peri-infarct zone after PT stroke. (A) (a-f)** Representative micrographs of BrdU and glial cell marker (Iba1 and GFAP) staining in the peri-infarct zone 6 days after PT stroke. **(B) (a, b)** The numbers of double positive cells were quantified and compared between groups. Scale bars = 50 μm. Data are mean ± SE, N = 6-8. *P < 0.05 versus Ctl group, ^#^*P* < 0.05 versus PT group.

To determine the effects of cTBS treatment on gliogenesis after PT stroke, we co-stained for BrdU/Iba1 and BrdU/GFAP to identify newborn microglial and astroglial cells, respectively. As seen in **Figure 5A (a-c) and B (a)**, PT stroke-induced significant proliferation of microglia in the peri-infarct zone 6 days after stroke, while this response was significantly ameliorated in cTBS group animals, indicating that cTBS reduced the activation response of these cells. Likewise, PT resulted in an increased number of GFAP^+^/BrdU^+^ cells in the peri-infarct regions, suggesting astrogliogenesis associated, in part, with glial scarring. Intriguingly, astroglial proliferation was abated significantly in rats treated with cTBS **(Figure 5A (d-f) and B (b))**. Taken together, these findings indicate that early cTBS administration is effective in reducing abnormal microglia and astrocyte proliferation.

### cTBS treatment promoted oligodendrogenesis in the peri-infarct zone after PT stroke

**Figure 6:**
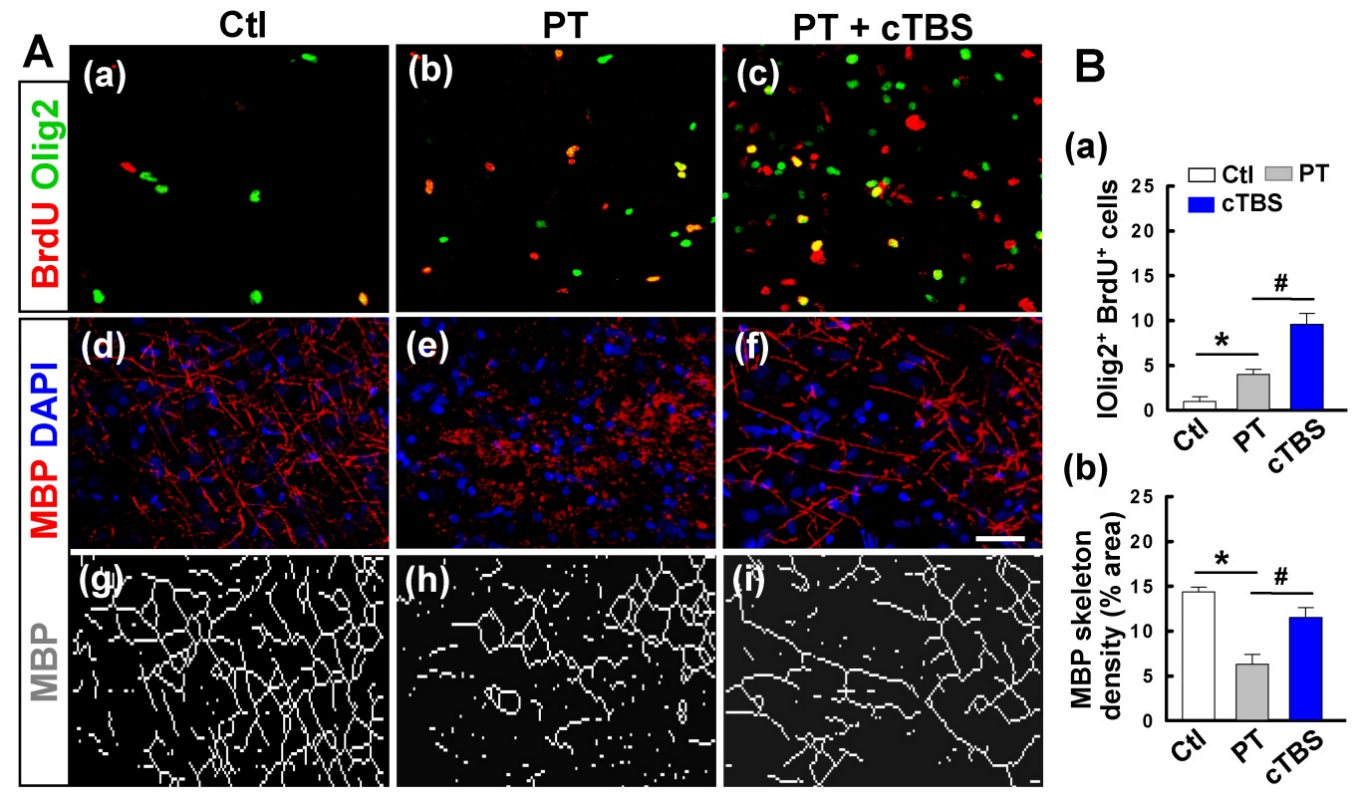
** cTBS treatment promoted oligodendrogenesis in the peri-infarct zone after PT stroke. A (a-c)** Double immunostaining with anti-BrdU (red) and anti-Olig2 (green) antibodies to identify proliferating cells and oligodendrocytes in the peri-infarct zone at day 6.** (d-i)** Typical fluorescence images of MBP-immunoreactivity (red) with the DAPI nuclear counterstaining (blue) 22 days after PT stroke. The network dense of MBP-positive structures, which represents myelination of nerves, was skeletonized and presented for a better view (g-i). **B (a, b)** The levels of oligodendrogenesis and MBP skeleton density were quantified and indicated. Scale bars = 50 μm. Data are mean ± SE, N = 6-8. *P < 0.05 versus Ctl group, ^#^*P* < 0.05 versus PT group.

Regeneration of mature myelinating oligodendrocytes is critical for myelin regeneration and functional recovery after ischemic stroke. Evidence suggests that improving endogenous oligodendrogenesis in the ischemic brain could facilitate brain repair processes and reduce neurological deficits 27. Thus, we sought to determine if cTBS affected post-stroke oligodendrogenesis. Brain sections were double-stained with BrdU and Olig2, as shown in **Figure 6A (a-c) and B (a)**, PT stroke-induced proliferation of oligodendrocyte precursors, and the level of proliferation was significantly increased in animals treated with cTBS. Next, at 22 days after PT stroke, we stained for MBP/DAPI to determine the degree to which cTBS promotes myelination, as seen in representative images in **Figure 6A (d-i)**. Skeletal density analysis of MBP staining found that PT group animals had significantly less MBP signal and thus less myelination. Animals treated with cTBS have significantly more myelination compared to control group animals, indicating that cTBS can promote myelination after stroke **(Figure 6B (b))**.

### cTBS enhanced FGF2 and BDNF expression in the peri-infarct zone after PT stroke

**Figure 7:**
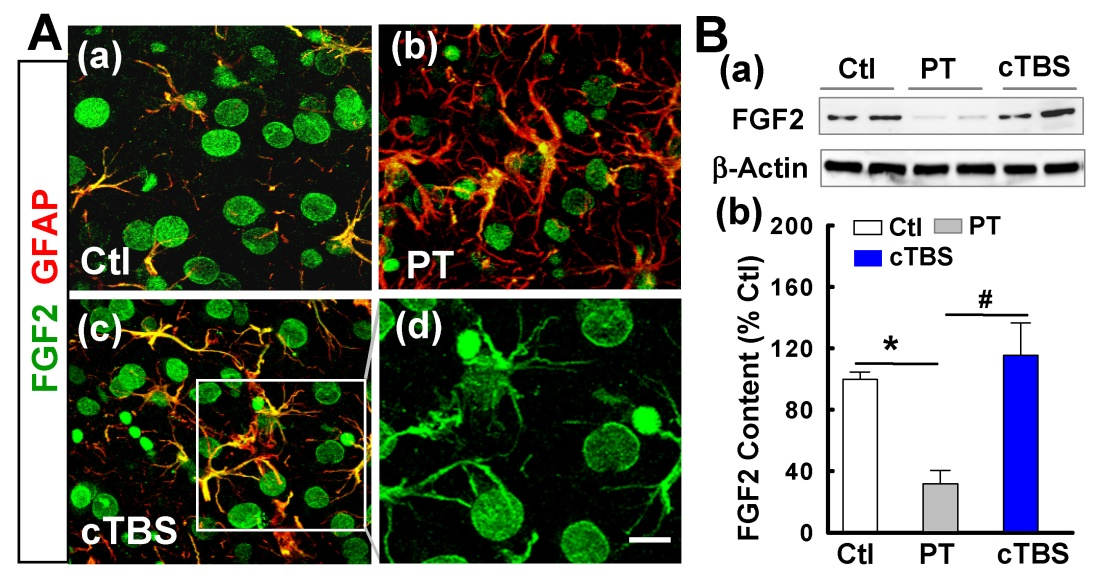
** cTBS enhanced FGF2 expression in the peri-infarct zone after PT stroke. (A) (a-d)** Double labeling of FGF-2 (green) and GFAP (red) in peri-infarct zone 6 days after PT stroke. Note FGF-2 immunoreactivity in astrocytes (merged yellow), whereas FGF2 appeared also associated with mature neurons and other cellular types (d, big round neuron-like cells and oligodendrocyte-like cells). **(B) (a,b)** Representative Western blots and quantitative analyses of FGF2 expression in peri-infarct proteins 6 days after PT stroke. Magnification: ×40, scale bar: 10 μm. Data are mean ± SE from 4-7 animals in each group. *P < 0.05 versus Ctl, ^#^*P* < 0.05 versus PT group without cTBS treatment.

Naive astrocytes shed extracellular vesicles containing large amounts of neuroprotective compounds, including the trophic factor FGF2 28. Such trophic factors, on the other hand, may also support neurogenesis after stroke 29. To determine whether cTBS can increase the levels of trophic factor FGF2, we co-stained for FGF2/GFAP. As shown in representative confocal images in** Figure 7A (a-d)**, FGF2 signal dramatically decreased in PT group animals compared to sham control. Animals in the cTBS group, however, have substantially increased colocalization of FGF2 and GFAP relative to those in the PT group, implying that cTBS may increase astrocytic FGF2 expression. For the cellular location, FGF2 appeared also associated with other cellular types, such as neuron-like (big round cells) and oligodendrocyte-like cells (**Figure 7A (d),** the separated FGF2 signal). To further verify the results of immunofluorescent staining, we detected the expression of FGF2 by Western blot. As shown in **Figure 7B (a, b)**, we also found that animals treated with cTBS showed a significant increase in FGF2 protein levels.

**Figure 8:**
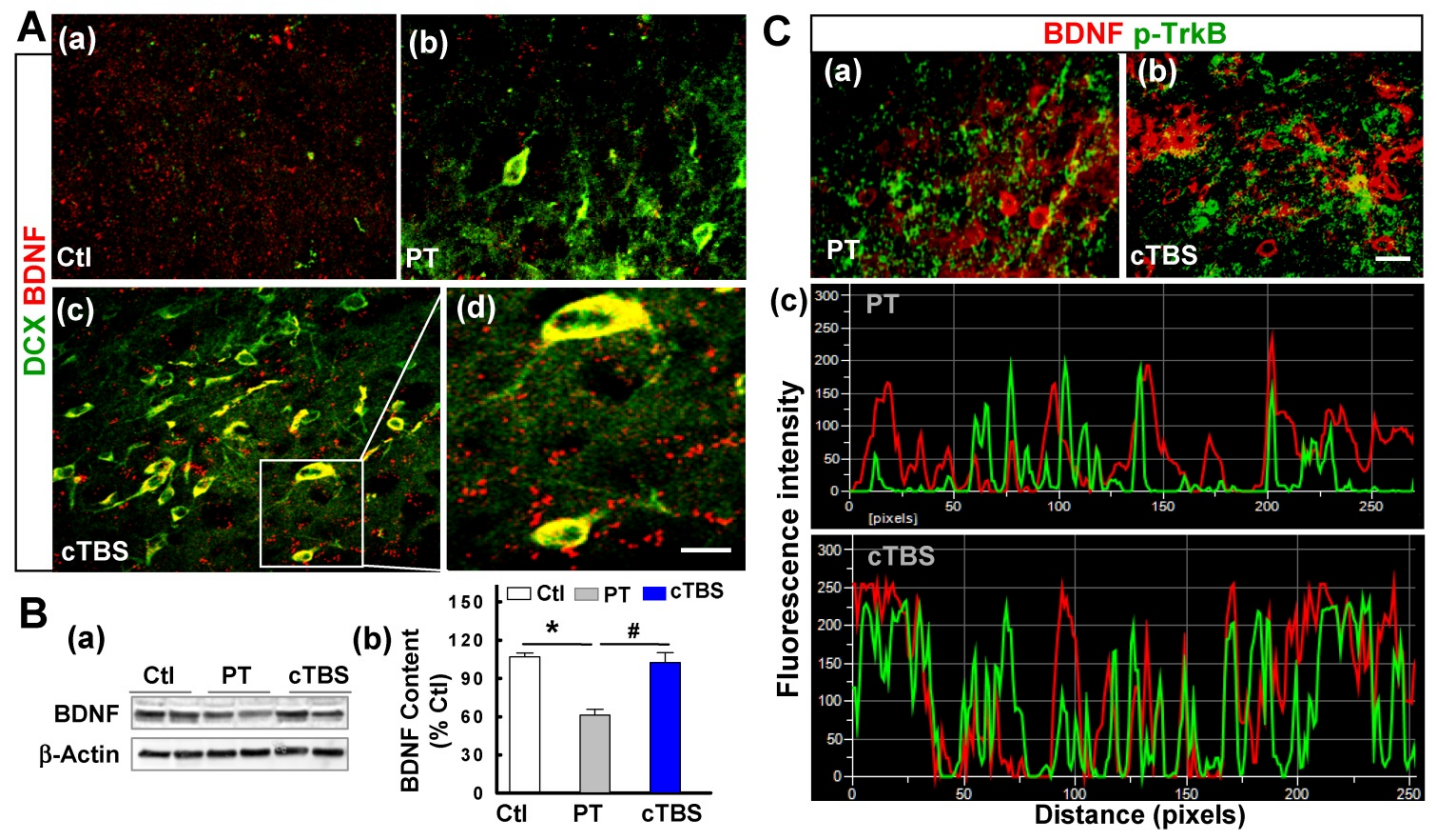
** cTBS enhanced BDNF expression in the peri-infarct zone after PT stroke. (A) (a-d)** Representative micrographs of DCX and BDNF double labeling in the peri-infarct area 6 days after PT stroke. The insert shows a zoomed view of DCX-positive neuroblast with high level of BDNF localization in cell soma, and the visible BDNF puncta with bright fluorescence spots around infarct border in cTBS-treated group. **(B) (a, b)** Representative Western blots and quantitative analyses of BDNF expression in peri-infarct proteins 6 days after PT stroke. **(C) (a,b)** Representative co-staining of BDNF (red), and p-TrkB (green) in the peri-infarct area 6 days after PT stroke. **(c)** Reprehensive fluorescence intensity profiles and the different degree of co-localization calculated on BDNF/p-TrkB co-immunostained images. Note the fluorescence peaks in cTBS group are higher and more closely associated compared to those in PT groups. Magnification: ×40, scale bar: 10 μm. Data are mean ± SE from 4-7 animals in each group. *P < 0.05 versus Ctl, ^#^*P* < 0.05 versus PT group without cTBS treatment.

BDNF is a trophic growth factor that supports the survival of immature neurons and oligodendrocyte progenitor cells after stroke 17. Next, we further investigated whether cTBS affects the level of BDNF. Representative confocal images double-stained with BDNF/DCX at day 6 after PT stroke suggested that cTBS augmented the expression of BDNF localized in the cell soma of peri-infarct DCX-positive neuroblast after PT stroke **(Figure 8A (a-d))**. As quantified via Western blot in **Figure 8B (b)**, total BDNF content was downregulated in PT group animals compared to control group. However, animals treated with cTBS had significantly more BDNF signal than PT animals. Finally, to determine whether cTBS can increase colocalization of BDNF and its receptor TrkB, we co-stained for BDNF and p-TrkB (phosphorylated tyrosine 816 residue of active TrkB receptor) in the peri-infarct region. Representative images **(Figure 8C (a, b))** and analysis **(Figure 8C (c))** showed a significantly increased colocalization and amount of overlapping fluorescent signal in cTBS animals compared to the PT group. Overall, these results further emphasize the role of specific trophic factors in cTBS-induced neurogenesis.

### cTBS treatment enhanced functional sensorimotor performance after PT stroke

**Figure 9:**
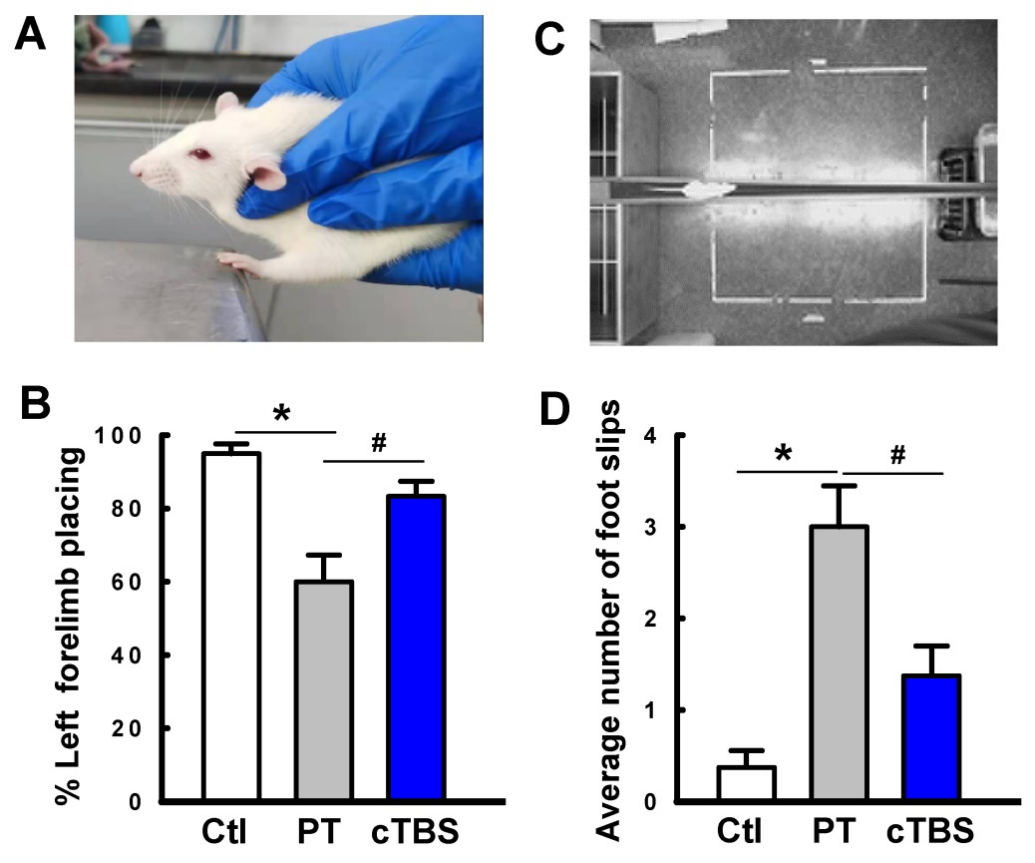
** cTBS treatment enhanced functional performance after PT stroke.** The animal's behavioral outcome were measured by forelimb placing test **(A, B,** for assessing forelimb function) and balance beam test (**C, D,** for the assessment of motor coordination) on day 22 following PT stroke. Representative images of forelimb placement and beam walking tests are shown (**A, C**). The percent successful left forelimb placing and average number of foot slips were analyzed and compared. Data are mean ± SE (N = 6-9). *P < 0.05 versus Ctl. ^#^*P* < 0.05 versus PT group without cTBS treatment.

**Figure 10:**
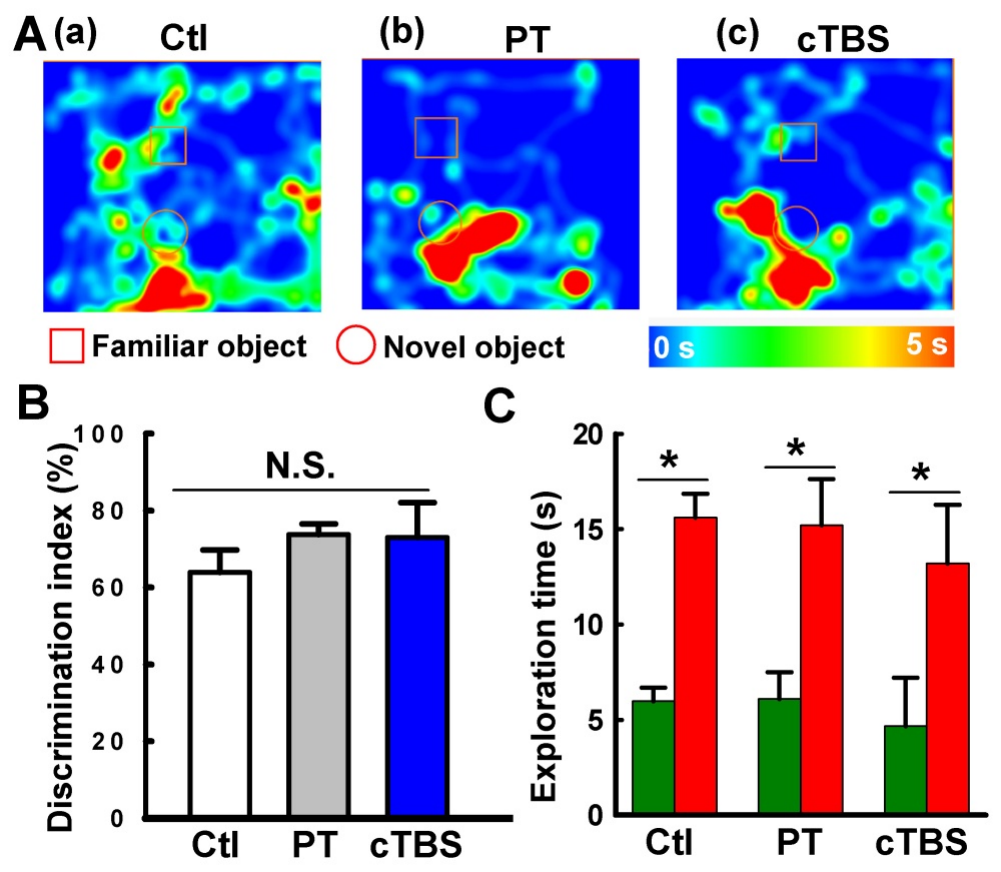
** Effects of PT stroke and cTBS treatment on learning and cognitive recognition memory. (A)** Novel object recognition tests on days 21 and 22 after PT stroke were conducted to test the learning and recognition memory. **(a-c)** Representative heat map of the animal's head was presented during exploration of the familiar object and a novel object. **(B)** Discrimination index were analyzed. **(C)** The time spent on exploring the familiar (dark green) and novel object (red). Data are mean ± SE (N = 6-9). *P < 0.05 between groups. N.S.: not statistically significant.

Stroke survivors typically undergo varying degrees of motor deficits 30. To determine whether cTBS treatment can improve functional performance impairments induced by PT stroke, animals underwent behavioral testing 22 days after PT. The forelimb placement test was performed to measure motor deficits. As seen in **Figure 9A&B**, PT animals suffered apparent deficits in left (contralateral) forelimb placement, which was significantly improved in animals treated with cTBS. Furthermore, locomotor coordination was examined using the balance beam test. Animals undergoing PT stroke had significantly more foot slips than control animals when crossing the beam **(Figure 9C&D)**. Notably, cTBS strikingly reduced the number of foot faults during crossing, indicating amelioration of motor impairments. Taken together, our findings show that cTBS treatment dramatically mitigates behavioral deficits following ischemic stroke. In addition, we examined the effects of PT stroke and cTBS treatment on learning and cognitive recognition memory, as depicted in **Figure 10**. However, we did not find significant changes in the learning and recognition memory tests among Ctl, PT and cTBS groups, suggesting cognitive function was not severely affected in this PT stroke model.

### Inhibition of neurogenesis by TMZ attenuated long-term survival of newborn neurons and functional recovery conferred by cTBS

**Figure 11:**
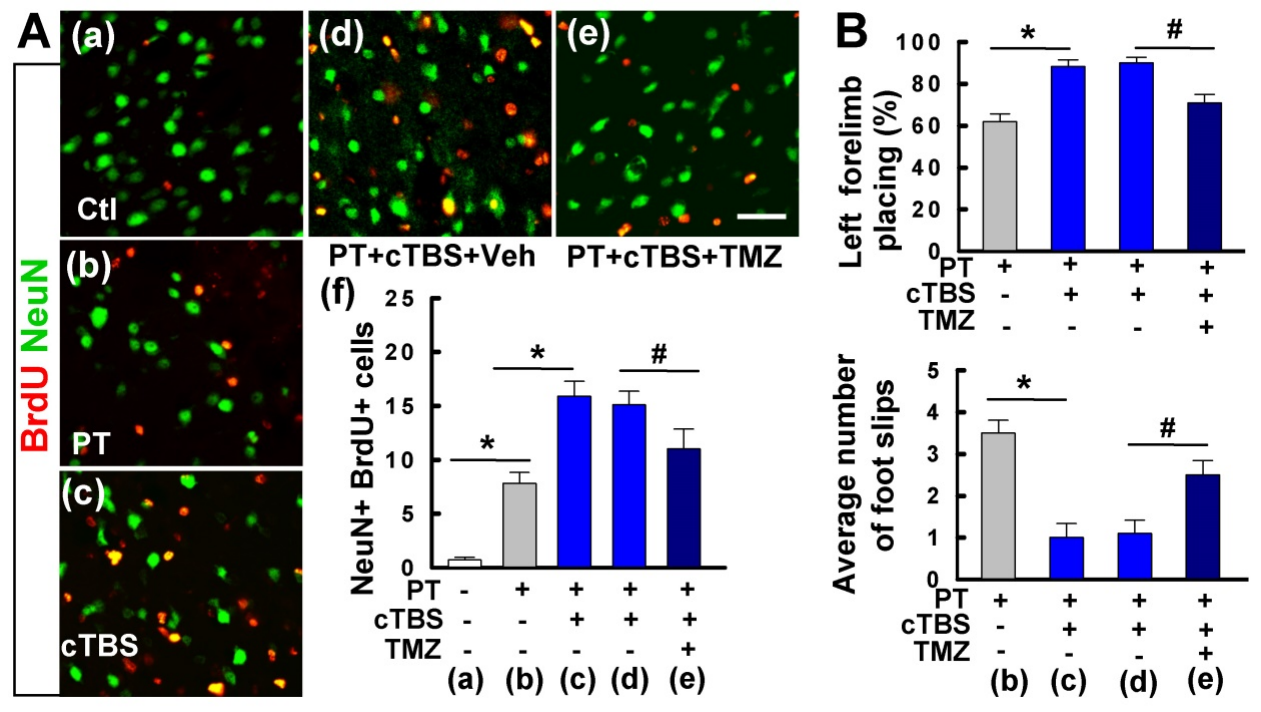
** Delayed cTBS treatment improved long-term survival of newly generated neurons and functional recovery, an effect was reversed by temozolomide. (A) (a-e)** cTBS was applied to the rats one week after PT stroke for 6 days. Representative confocal microscope images of BrdU (red) and NeuN (green) in the peri-infarct area 5 weeks after PT stroke. Total numbers of double-labeled cells from each group were counted and analyzed **(f)**. Temozolomide (TMZ) or vehicle (Veh) was administrated to cTBS treated-rats with osmotic mini-pumps for 7 days as described in the methods.** (B)** Motor function was measured by forelimb placing test (% of left forelimb placing) and balance beam test (average number of foot slips) 5 weeks after PT stroke. Scale bars = 50 μm. Data are mean ± SE (N = 8-10). *P < 0.05 versus PT or Ctl group, ^#^*P* < 0.05 versus cTBS or vehicle group.

We also examined the long-term effects of cTBS when applied at a later time point following ischemic stroke. As shown in** Figure 11A(a-f)**, when administered one week after PT stroke, cTBS treatment (last for 6 days) also significantly enhanced the counts of NeuN^+^/BrdU^+^ cells, suggesting that both early and late application of cTBS is effective in promoting post-stroke neurogenesis. To be noted, this treatment profile was also effective in improving motor function, in a long-term manner, as evidenced by the forelimb placing test and balance beam test 5 weeks after PT stroke (**Figure 11B**). To determine whether cTBS facilitated functional recovery was associated with improved neurogenesis, we administered TMZ to the animals with cTBS treatment. TMZ has been used to suppress adult neurogenesis in previous reports 31, 32. As illustrated in **Figure 11A(e, f)**, TMZ administration could significantly attenuate cTBS-improved neurogenesis as well as the long-term functional recovery, suggesting an additional role of cTBS in repairing the stroke brain by enhancing peri-infarct neurogenesis.

## Discussion

Stroke, particularly ischemic stroke, represents a significant public health problem and a leading cause of disability worldwide. However, treatment options are still limited, leaving debilitating consequences for most patients. As the population ages, the incidence of stroke continues to increase annually, and a novel therapeutic approach is urgently needed to improve stroke outcomes for patients 33, 34. The current study used a photothrombosis-induced ischemic stroke model, and we observed a positive effect of cTBS on the enhancement and maintenance of neurogenesis and oligodendrogenesis following stroke. Furthermore, mechanistic studies revealed that such effects might be supported by the release of trophic factors associated with astrocytes and DCX-positive neuroblasts. These findings highlight the potential of using cTBS as an adjunctive treatment strategy for stroke.

Adult neurogenesis involves the proliferation, survival, differentiation, and integration of newborn neurons into existing neural networks 35. It is well established that this process occurs in certain regions of the brain, primarily in the lateral ventricular SVZ and in the SGZ of the dentate gyrus 36, 37. Although adult neurogenesis has remained a controversial topic, higher levels of neurogenesis markers in SVZ and ischemic penumbra surrounding cerebral cortical infarcts have been observed in patients (both young and elderly) died from acute stroke than in individuals died normally 38-40. Furthermore, preclinical studies have suggested that interventions that modulate adult neurogenesis have some therapeutic potential for stroke 26, 41. More specifically, adult neural stem cells in the SVZ and SGZ can be activated in response to stroke, proliferate to produce neural progenitor cells, and migrate to the peri-infarct region, which is critical for brain repair processes during stroke recovery 26, 41, 42. Indeed, by retroviral tracing of NSCs, Ohira K et al. found that cortical neurogenesis induced by cerebral ischemia originated mainly from NSCs generated in the cortex, and only a small amount of NSCs originating from the SVZ migrated and eventually formed functional mature neurons in damaged areas 43. In our current study, we did not check the SVZ and SGZ areas for neurogenesis because of the nature of this PT stroke model. This animal model typically induces ischemic infarct within the desired right sensorimotor neocortex. Nevertheless, we cannot rule out neurogenesis and potential functional roles from the SVZ and SGZ areas following ischemic damage and cTBS treatment.

Preclinical studies have revealed that strategies aimed to enhance neurogenesis, such as cell transplantation therapy, facilitated motor function recovery after stroke 44-46. Recent findings indeed suggest that neurogenesis can also occur in the infarct and surrounding areas, including the PT stroke model 10. Although neurogenesis is usually connected with cognitive function, peri-infarct cortical neurogenesis contributes significantly to motor recovery 47, 48. In our current study, we also performed novel object recognition tests to examine the learning and recognition memory functions following PT stroke and with cTBS treatment. Intriguingly, we did not find significant effects of PT stroke and cTBS treatment on the learning and cognitive recognition memory (see **Figure 10**), implying that this PT stroke does not cause cognitive deficits, and that cTBS-improved neurogenesis in the peri-infarct zone has no significant effects on learning and memory in PT stroke.

Several lines of evidence highlighted that selective ablation of post-stroke neurogenesis interferes with neurological recovery, suggesting this endogenous process is necessary for brain recovery 49-51. Unfortunately, this endogenous neurogenesis occurs slowly, and the newborn neurons usually struggle to survive over the long term, possibly due to the lack of trophic factors in their microenvironment and chronic inflammatory response. Hence, amplifying endogenous neurogenesis and protecting the survival of the newborn neurons may represent a promising therapeutic approach for stroke. Interestingly, certain neurotrophic factors primarily secreted by glial cells, such as BDNF and FGF2, have been shown to support neurogenesis 52-55. In experimental animal models, BDNF and FGF2 have been reported to regulate neurogenesis after ischemic stroke and are associated with improved functional outcomes, including motor and cognitive function 56, 57. Furthermore, it has been suggested that BDNF may be a promising predictor for motor outcomes in patients with subacute stroke 58. Taken together, these reports highly support our findings of the pivotal role of cTBS in enhancing neurotrophic factors and neurogenesis to facilitate functional recovery after stroke.

TBS is a variant of TMS that uses 50 Hz triplet pulse burst with a 200 ms inter-pulse interval. Two primary TBS protocols have been developed: Continuous TBS (cTBS) and intermittent TBS (iTBS), which normally attain the capacity to increase and decrease cortical excitability, respectively 19. Notably, the after-effect of rTMS is different in human subjects, and emerging research reveals that the effect of rTMS also highly depends on the interval between bursts and the total length of conditioning 19. Additionally, most studies have shown that single or short-term cTBS (3 × 50 Hz pulses repeated every 200 ms) can induce LTD. In this study, we applied a 5-min cTBS paradigm for 6 days, whether this paradigm induced inhibitory or facilitatory alterations in the cortex remains to be determined. Given that stroke could lead to the disruption of the equilibrium of cortical excitability between the two hemispheres. A better understanding of this information will shed new light on the mechanisms behind the neuroprotective effects of cTBS and contribute to personalized treatment programs.

A previous study has shown that compared with conventional TMS, TBS dramatically induces genes involved in angiogenesis, cellular repair, neuroprotection, and neuroplasticity and ameliorates behavioral deficits induced by stroke 59. In this study, we found that cTBS increased the neural progenitor cell pool and the number of newly generated neurons in the peri-infarct zone after PT stroke, as evidenced by increased cell numbers in Nestin/SOX2 and DCX/BrdU double staining. This is consistent with literature showing that TMS enhances neurogenesis after stroke 60, 61. In addition, we further explored whether cTBS could promote the survival of newborn neurons. Using Tunel/SOX2, TUNEL/BrdU/NeuN, and NeuN/BrdU co-staining, we observed that cTBS attenuated apoptotic cell death of neural progenitor cells and newborn neuronal cells in the peri-infarct area, and increased the number of newly generated neurons at 22 days after PT stroke. Collectively, these results clearly indicate that cTBS effectively enhances and sustains neurogenesis following ischemic stroke.

Our previous studies have shown the therapeutic potential of cTBS stimulation in the early post-stroke period. Early rTMS treatment improved synaptic loss in peri-infarct cortical regions, attenuated neuronal degeneration, pro-inflammatory factor overproduction and oxidative damage, facilitated and sustained post-ischemic angiogenesis, and alleviated blood-brain barrier dysfunction 18, 21. These findings suggest a multifaceted role for cTBS in promoting stroke recovery. Herein, we report that cTBS effectively promoted neurogenesis in the peri-infarct cortical region. To be noted, the administration of TMZ, a drug that has been previously used to inhibit adult neurogenesis, could significantly limit the enhanced neurogenesis and functional recovery conferred by cTBS. Therefore, this work provides new insights into the mechanisms by which cTBS contributes to stroke recovery.

Astrocytes, oligodendrocytes, and microglia are the primary glial cells in the central nervous system and affect neural plasticity and repair in distinct ways 62, 63. When an ischemic stroke occurs, astrocytes and microglia are activated to exert both detrimental and neuroprotective mediators 62. Astrocytes and microglia, the brain's resident macrophages, are implicated in post-stroke immune regulation. In ischemic stroke, glial cell activation and reactive glial proliferation contribute to limiting brain injury and restoring CNS homeostasis, but aberrant gliogenesis may be associated with aggravated tissue damage and neurological dysfunction 17, 64. It has been suggested that inhibiting reactive glial cell activation after insult may facilitate neural recovery 65, 66.

On the other hand, in response to ischemia stroke, reactive astrocytes produce various neurotrophic factors including BDNF 67, FGF2 68, and glial-derived neurotrophic factor 69, 70 to protect neurons, thus contributing to the restoration of central nervous system homeostasis. Furthermore, it has been proposed that these astrocytes may support neuroblasts migrating to the infarcted brain area 12, 71. Activated astrocytes, however, also release inflammatory cytokines that induce neural tissue damage, and the formation of glial scarring can impede neuronal reconnection and extension 62. In this study, we found that treatment with cTBS mitigated gliogenesis to a relative low level in the peri-infarct zone after PT stroke. It is noteworthy that cTBS treatment also promotes the expression of FGF2 and BDNF, suggesting cTBS may facilitate glial cells conversion to anti-inflammatory phenotype, similar to our previous observations showing that cTBS could induce a shift from A1 to A2 in astrocytic phenotypes 18.

Correspondingly, oligodendrogenesis, the formation of new oligodendrocytes, is a critical brain repair process following stroke 17, 27. Stroke can lead to the loss of oligodendrocytes and their myelin, thereby impairing axonal function. Since mature oligodendrocytes do not proliferate in the adult brain and damaged oligodendrocytes are unable to form new myelin sheaths, new oligodendrocytes are needed to form myelin sheaths to germinate axons 27, 72. Studies on animal models of ischemic stroke have shown that enhanced endogenous oligodendrogliosis promotes repair processes in the brain and reduces neurological deficits 73-75. Using BrdU/Olig2 co-staining, we reported that cTBS positively affects the induction of oligodendrogenesis after PT stroke and maintains myelination. Given that previous studies showed that BDNF and FGF2 could promote oligodendrogenesis in multiple physiological and pathological conditions 76, 77, these positive effects may also be partially attributed to the increased release of certain trophic factors.

Moreover, it has been reported in clinical and animal studies that stroke often leads to long-term functional performance impairment 78-80. Therefore, strategies to improve functional outcomes are the focal point for stroke treatment. On day 22 after PT stroke, we used the forelimb placement test and balance beam test to analyze functional performance in animals. Intriguingly, treatment with cTBS remarkably reversed the abnormalities in motor functions. Taken together, these data indicate that cTBS may hold significant promise as a noninvasive tool to facilitate functional recovery from stroke.

Although there are promising results, more variables should be considered before cTBS can be developed as a therapeutic measure for stroke. Our study points out that neurogenesis may be an important target for cTBS. Nevertheless, whether adult neurogenesis exists in humans is still debatable. Therefore, the clinical value of therapies aimed to promote adult neurogenesis remains to be established. Differences in the onset of cTBS administration, the location of stimulation, and the duration of treatment may lead to distinct functional outcomes. In addition, while promoting endogenous neurogenesis represents a potential therapeutic target, aberrant neurogenesis after stroke may also lead to cognitive impairment 81. Further work will be needed to establish the optimal cTBS protocol and explore its safety range. Based on immunofluorescence staining analyses, our findings imply that early cTBS treatment promotes neurogenesis in the peri-infarct cortical region. However, the application of genetic genealogy tracking techniques will provide further reliable evidence, which is a limitation of the current study. Moreover, in this study, we applied a 6-day cTBS treatment procedure. Whether the extended treatment is more beneficial for neurogenesis and functional recovery after stroke remains to be determined. These issues will be our next goals in future studies. A more comprehensive understanding of the molecular mechanisms behind these effects will lead to more satisfying applications of this noninvasive technique.

In conclusion, the results presented in this study demonstrate that cTBS, when administered to the infarct hemisphere both 3 h and one week after PT stroke, efficiently promotes and sustains neurogenesis in the peri-infarct area. We also observed that cTBS significantly mitigated functional deficits after ischemic stroke. Strikingly, the inhibition of neurogenesis could limit these therapeutic benefits conferred by cTBS. Mechanistically, cTBS suppressed aberrant gliogenesis and promoted oligodendrogenesis in the peri-infarct zone, these effects may be partly due to increased release of trophic factors such as BDNF and FGF2. Taken together, although further study is still needed to elucidate the in-depth mechanism of neurogenesis induced by cTBS, these results collectively suggest that both early and delayed application of cTBS can positively affect post-stroke neurogenesis and neuronal survival, supporting its potential as a noninvasive therapeutic strategy for stroke recovery.

## Abbreviations

TMS: transcranial magnetic stimulation
cTBS: continuous theta-burst stimulation
BDNF: brain-derived neurotrophic factor
FGF2: fibroblast growth factor 2
PT: photothrombotic
NPCs: neural progenitor cells
tPA: plasminogen activator
SGZ: subgranular zone
SVZ: subventricular zone
